# Targeting Cathepsin Z suppresses endometriosis via the AMPK/mTOR-mediated autophagy and apoptosis axis

**DOI:** 10.3389/fmed.2026.1681467

**Published:** 2026-09-01

**Authors:** Mengjun Zhang, Jialin Wang, Haodi Yue, Zidi Zhang, Lindong Zhang, Xin Zhao

**Affiliations:** 1 Department of Gynecology, The Third Affiliated Hospital of Zhengzhou University, Zhengzhou, China; 2 Department of Orthopedics, The First Affiliated Hospital of Zhengzhou University, Zhengzhou, China; 3 Department of Center for Clinical Single Cell Biomedicine, Henan Provincial People's Hospital, People's Hospital of Zhengzhou University, Zhengzhou, China; 4 Department of Radiology, The Third Affiliated Hospital of Zhengzhou University, Zhengzhou, China; 5 Tianjian Laboratory of Advanced Biomedical Sciences, Institute of Advanced Biomedical Sciences, Zhengzhou University, Zhengzhou, China

**Keywords:** AMPK/mTOR signaling pathway, autophagy, cathepsins, endometriosis, therapeutic targets

## Abstract

**Background:**

The key biomarkers, therapeutic targets, and pathological mechanisms of endometriosis are urgently needed to be elucidated. The role of CTSZ in EM needs to be clarified, and the potential regulatory mechanisms and corresponding therapeutic drugs of CTSZ need to be explored to demonstrate the feasibility of CTSZ as a biomarker and therapeutic target.

**Methods:**

Based on transcriptome sequencing of local clinical tissue samples and public data, the key pathogenic gene (*CTSZ*) was screened out using machine learning algorithms (LASSO and SVM-RFE), and the expression of CTSZ was evaluated at multiple levels. The effects of knocking down CTSZ on the proliferation and migration of endometriosis cell lines (12Z) were assessed. The effects of knocking down CTSZ on apoptosis, autophagy and AMPK/mTOR signaling pathways were predicted and investigated.

**Results:**

*CTSZ* was found to be abnormally overexpressed in sequencing data, endometriosis clinical tissue samples, and corresponding cell lines (12Z), suggesting it is a potential pathogenic gene. Knockdown of CTSZ significantly inhibited the proliferation and migration of 12Z. CTSZ may be related to key processes and proteins of apoptosis, autophagy and AMPK/mTOR signaling pathways. Knockdown of *CTSZ* significantly promoted apoptosis and autophagy by activating AMPK and subsequently inhibiting mTOR signaling.

**Conclusion:**

CTSZ is a potential pathogenic gene and therapeutic target. Its knockdown could activate AMPK and subsequently inhibit mTOR signaling, thereby inducing autophagy and synergistically promoting apoptosis, inhibiting proliferation and migration, and ultimately inhibiting endometriosis.

## 1 Introduction

Endometriosis (EM) causes more than 100 million women around the world to suffer from pelvic pain and infertility, which is in urgent need of improvement (1, 2). Surgery is an important means to diagnose and treat EM, but it requires high technical requirements and may recur after surgery (3). The main reason for the lack of non-invasive early diagnosis and treatment methods is that the key pathological mechanisms of the occurrence and development of EM are not yet fully understood (4, 5). Therefore, in-depth exploration of the key biomarkers and potential molecular targets of EM will help clinical transformation and development of non-invasive diagnosis and treatment methods, thereby reducing the health and economic burden of EM, a global disease.

Cathepsin is a key enzyme in lysosomes, acting as a “cell molecular scissors” to maintain protein homeostasis (6). Its functional imbalance may cause cancer (7, 8), inflammation (9, 10), and even EM (11), and has therefore become a golden target for drug development (12). Cathepsin B (CTSB) (11) and Cathepsin G (CTSG) (13–15) have both been classically associated with extracellular matrix degradation and local tissue remodeling in EM. Some studies have even used these traditional cathepsins as targets for inhibition, thereby delaying the disease progression of EM (16). However, distinct from the conventional proteolytic roles of CTSB and CTSG, Cathepsin Z (CTSZ) possesses unique functional characteristics. It is not only related to the occurrence and poor prognosis of prostate cancer (17), but also participates in the regulation of neuroinflammation (18) and acute skin delayed hypersensitivity reactions (19), and affects vascular endothelial function and angiogenesis (20, 21). Given that angiogenesis and neuroinflammation are critical drivers of lesion survival and pain in EM, CTSZ may exert more complex pathogenic effects than its family members. Therefore, considering that EM, a benign disease similar to tumors, is closely related to inflammation and angiogenesis, the role of CTSZ in EM and its feasibility as a therapeutic target need to be explored urgently.

The inhibition of autophagy can regulate lipid metabolism through lysosomes, thereby regulating the synthesis of sex steroid hormones such as estrogen and progesterone, thereby promoting the occurrence of EM (22, 23). The increase in estrogen receptors will inhibit cell apoptosis, thereby exacerbating the progression of EM (23). These studies all suggest that autophagy, apoptosis, and lysosomes may have a profound impact on EM. At the same time, studies have also shown that the stability of lysosomes can be destroyed by B10, which regulates CTSZ and promotes the induction of autophagy and apoptosis (24). Therefore, exploring the connection between CTSZ, apoptosis, and autophagy will help understand the pathological mechanism of EM and develop therapeutic targets.

Therefore, this study attempted to combine local clinical tissue samples, transcriptome sequencing, public data, machine learning, and multi-level experiments to deeply explore and elucidate the complex crosstalk and molecular pathways of EM, CTSZ, apoptosis, and autophagy, and to demonstrate the feasibility of CTSZ as a potential noninvasive biomarker and therapeutic target, ultimately improving the diagnosis and treatment dilemma of EM.

## 2 Materials and methods

### 2.1 Data collection and target gene screening

Public transcriptomic datasets related to endometriosis (GSE51981 and GSE7305) were acquired from the Gene Expression Omnibus (GEO) database. Concurrently, an initial local clinical cohort comprising ectopic endometriosis tissues (specifically identified as ovarian endometriomas) (n = 5) and normal control endometrium (n = 5) was collected from patients undergoing laparoscopic surgery at the Third Affiliated Hospital of Zhengzhou University. The study protocol was strictly reviewed and approved by the Ethics Committee of the Third Affiliated Hospital of Zhengzhou University (Approval No. 2024–174), and written informed consent was obtained from all participants prior to surgery. Both the public data and the initial local RNA sequencing data were utilized for differential expression analysis and machine learning-based key gene screening. Notably, for subsequent experimental validations including RT-qPCR, Western blotting, and immunohistochemistry (IHC), the local clinical cohort was expanded to encompass a total of 6 representative patient pairs (n = 6) to ensure broader biological reproducibility.

#### 2.1.1 Machine learning-based feature selection

To robustly identify and validate the key diagnostic features, two classical machine learning algorithms were concurrently employed utilizing R software (version 4.2.0). The Least Absolute Shrinkage and Selection Operator (LASSO) logistic regression was executed via the “glmnet” R package. The optimal penalty parameter (lambda) was determined through a 10-fold cross-validation approach to prevent model overfitting. Concurrently, the Support Vector Machine-Recursive Feature Elimination (SVM-RFE) algorithm was implemented using the “e1071” and “caret” R packages. SVM-RFE was utilized to rank features and compute classification errors via 5-fold cross-validation. The overlapping features identified by both algorithms were selected for subsequent downstream analyses, ultimately screening out the core target gene, *CTSZ*. Subsequently, to quantitatively evaluate the diagnostic performance of the identified core biomarker, Receiver Operating Characteristic (ROC) curve analysis was conducted, and the Area Under the Curve (AUC) was calculated.

#### 2.1.2 Downstream bioinformatics and connectivity map (cMAP) analysis

For the identified key gene (*CTSZ*), its subcellular localization was evaluated via the Human Protein Atlas (HPA). Protein–protein interaction (PPI) networks were constructed to elucidate the functional interactions between CTSZ and autophagy-related proteins. Subsequently, Gene Ontology (GO) and Kyoto Encyclopedia of Genes and Genomes (KEGG) enrichment analyses were performed to decipher potential biological functions and molecular regulatory pathways. Finally, to identify potential therapeutic compounds capable of targeting the disease signature, the Connectivity Map (cMAP) database was queried via the CLUE.io platform (https://clue.io/). Briefly, the top 150 positively correlated genes and top 150 negatively correlated genes of *CTSZ* were inputted as the “up-regulated” and “down-regulated” signatures, respectively. Compounds yielding a highly negative enrichment score (< −90) were considered as potential therapeutic candidates. Further experimental validations (RT-qPCR, Western blotting, and IHC) were subsequently conducted based on these bioinformatics findings.

### 2.2 Cell culture and gene knockdown

The primary cell lines utilized in this study encompassed immortalized human endometrial epithelial cells (T-hEECs) and human immortalized endometriosis cells (12Z). The procurement, cultivation, and passaging of these cell lines were executed strictly in accordance with standardized protocols described in previous studies (25).

To achieve transient gene silencing, the 12Z cell line was transfected with three distinct candidate small interfering RNAs (designated as si-429, si-635, and si-727) targeting different regions of the *CTSZ* gene, alongside a scrambled non-targeting negative control (si-NC). All custom siRNA oligonucleotides were synthesized and purified by GenePharma (Shanghai, China). The detailed sequence configurations for these duplexes were as follows: (1) si-635: sense (5′-GGGACAUGCAAUGAAUUCATT-3′) and antisense (5′-UGAAUU CAUUGCAUGUCCCTT-3′); (2) si-429: sense (5′-CGGAUC GGAUCAACAUCAATT-3′) and antisense (5′-UUGAUG UUGAUCCGAUCCGTT-3′); (3) si-727: sense (5′-GAUGAUGGCA GAAAUCUAUTT-3′) and antisense (5′-AUAGAUUUCUGCCAUC AUCTT-3′); and (4) si-NC: sense (5′-UUCUCCGAACGUGUCAC GUTT-3′) and antisense (5′-ACGUGACACGUUCGGAGAATT-3′).

Transfections were mediated using Lipofectamine reagent (Invitrogen, Carlsbad, CA, USA) in serum-free OPTI-MEM basal medium. Cells were harvested 24 h post-transfection for subsequent validation. The comparative knockdown efficiencies of these constructs were systematically evaluated via RT-qPCR and Western blotting. Based on its superior gene silencing capability, si-727 was uniquely selected as the optimal construct for all subsequent cytological and functional downstream experiments to explore the biological effects of *CTSZ* ablation.

### 2.3 RNA extraction and RT-qPCR

To evaluate the mRNA expression levels of *CTSZ*, RT-qPCR was performed on both the local clinical tissue cohorts and the 12Z cell line across various transfection groups. Total RNA was extracted from the cells and tissues using the Total RNA Kit I (Omega Bio-tek, Norcross, GA, USA) strictly following the manufacturer’s standard protocols described in previous studies (25). The concentration and purity of the isolated RNA were quantified spectroscopically.

Subsequently, complementary DNA (cDNA) was synthesized using the NovoScript Plus All-in-one 1st Strand cDNA Synthesis SuperMix (Novoprotein, Suzhou, China). Quantitative real-time PCR was then conducted utilizing a standard SYBR Green qPCR Master Mix on a standard PCR thermocycler. The specific primer sequences were as follows: *CTSZ* forward (5′-CAGCGGATCTGCCCAAGAG-3′) and reverse (5′-CGATGACGTTCTGCACGGA-3′); *GAPDH* (utilized as the endogenous reference) forward (5′-CAAGGTCATCCATGAC AACTTTG-3′) and reverse (5′-GTCCACCACCCTGTTGC TGTAG-3′). The thermal cycling conditions were set as follows: initial denaturation at 95 °C for 10 min, followed by 40 cycles of denaturation at 95 °C for 10 s, and annealing/extension at 60 °C for 30 s. The relative mRNA expression levels were calculated using the standard 2^−ΔΔ*Ct*^ method.

### 2.4 Immunohistochemistry

Immunohistochemistry (IHC) was performed on the local clinical tissue cohorts to evaluate the spatial protein expression and localization of CTSZ. The experimental procedures, encompassing formalin-fixed paraffin-embedded (FFPE) tissue sectioning, dewaxing, rehydration, antigen retrieval, and endogenous peroxidase blocking, were executed strictly in accordance with standard protocols validated in previous literature (25).

Sections were incubated with the anti-CTSZ primary antibody at 4 °C overnight, followed by incubation with appropriate horseradish peroxidase (HRP)-linked secondary antibodies at room temperature. Immune complexes were visualized using a 3,3′-diaminobenzidine (DAB) substrate kit, and slides were counterstained with hematoxylin. To ensure absolute experimental transparency, a detailed description of the antibody specifications—including clone numbers, manufacturers, and working dilutions—is comprehensively provided in Supplementary Table S1.

### 2.5 Western blotting

Total protein extraction from both clinical tissue specimens and cultured 12Z cell lines across different transfection groups was performed using RIPA lysis buffer supplemented with protease and phosphatase inhibitors. The western blotting assays followed standard methodologies outlined in established protocols (25). Equal amounts of protein samples (30 μg per lane) were separated via 10% or 12% SDS-PAGE gels and subsequently electrotransferred onto polyvinylidene difluoride (PVDF) membranes.

After blocking with 5% non-fat milk or bovine serum albumin (BSA) at room temperature for 1 h, the membranes were probed with specific primary antibodies against apoptotic, autophagic, and AMPK/mTOR pathway-related targets at 4 °C overnight. Membranes were then washed and incubated with secondary antibodies. Protein bands were detected using an enhanced chemiluminescence (ECL) detection system. The comprehensive directory of all primary and secondary antibodies, as well as the essential detection reagents utilized in this study (including their respective catalogue numbers and dilution ratios), is meticulously documented in Supplementary Table S1.

### 2.6 CCK-8 assay

Cell viability was dynamically monitored utilizing a Commercial Cell Counting Kit-8 (CCK-8; Beyotime, Shanghai, China, Cat# C0037) in accordance with established standard procedures (25). Briefly, at 24 h post-transfection with si-*CTSZ* or si-NC, 12Z cells were harvested and seeded into 96-well plates at an initial density of 3 × 10^3^ cells per well in 100 μL of complete medium. The plates were incubated under standard humidified conditions (37 °C, 5% CO₂) for 0, 24, 48, and 72 h. At each designated time point, 10 μL of CCK-8 reagent was introduced into each well, followed by an additional incubation duration of 2 h. The optical density (OD) at a wavelength of 450 nm was determined utilizing a microplate reader.

### 2.7 EdU immunofluorescence

Cell proliferation was quantitatively evaluated via an EdU Proliferation Kit (RiboBio, Guangzhou, China, Cat# C10310-1) following the manufacturer’s optimization guidelines. Transfected 12Z cells were inoculated into 96-well plates at a density of 1 × 10^4^ cells/well and allowed to adhere overnight. Cells were then exposed to 50 μM of EdU labeling solution and cultured for a duration of 2 h at 37 °C. Subsequently, cells underwent fixation with 4% paraformaldehyde for 15 min and permeabilization with 0.5% Triton X-100 for 10 min at room temperature. Apollo reaction cocktail and Hoechst 33342 solution were sequentially introduced for nuclear counterstaining. Proliferation efficiency was quantified as the percentage of EdU-positive cells captured under a fluorescence microscope.

### 2.8 Wound healing assay

For horizontal migration analysis, 12Z cells from different transfection groups were inoculated into 6-well plates at a density of 3 × 10^5^ cells/well and cultured until achieving a confluent monolayer (approximately 90–100% confluence). A linear wound track was generated across the monolayer utilizing a sterile 200 μL pipette tip. Cellular debris was removed by washing thrice with sterile PBS. The cells were then cultured in serum-free medium for a duration of 24 h to eliminate the confounding effect of cell proliferation. Visual scratch fields were captured at 0 and 24 h utilizing an inverted microscope, and the migration capacity was determined by calculating the wound healing rate.

### 2.9 Transwell migration assay

Vertical cell migration was determined using 24-well Transwell chambers (8 μm pore size; Corning, NY, USA) according to standard protocols (25). Transfected 12Z cells were harvested, resuspended in serum-free medium, and seeded into the upper chamber at a density of 5 × 10^4^ cells per chamber (in 200 μL volume). Concurrently, 600 μL of complete medium supplemented with 10% fetal bovine serum (FBS) was introduced into the lower chamber to serve as a chemoattractant. Following a treatment duration of 24 h at 37 °C, non-migrating cells on the upper surface were meticulously removed with a cotton swab. Cells that successfully invaded the lower membrane surface were fixed with 4% paraformaldehyde for 20 min and stained with 0.1% crystal violet for 15 min. Migrated cells were photographed and counted in five randomly selected fields.

### 2.10 Clonogenic assay

To assess long-term colony-forming capabilities, 12Z cells were collected 24 h post-transfection, thoroughly dissociated, and seeded into 6-well plates at a low density of 1 × 10^3^ cells per well. The cells were cultured in complete medium at 37 °C for a duration of 14 days, with the culture medium replenished every 3 days. When visible colonies containing more than 50 cells emerged, the colonies were fixed with 4% paraformaldehyde for 15 min and visualized via staining with 0.1% crystal violet for 20 min. The number of colonies was quantified using ImageJ software.

### 2.11 Apoptosis flow cytometry

Quantitative evaluation of cell apoptosis was executed using an Annexin V-FITC/PI Apoptosis Detection Kit (Vazyme, Nanjing, China, Cat# A211) according to standard protocols. At 48 h post-transfection, 12Z cells were detached utilizing EDTA-free trypsin, harvested via centrifugation, and washed twice with ice-cold PBS. The cell pellet was resuspended in 100 μL of 1 × Binding Buffer, to which 5 μL of Annexin V-FITC and 5 μL of Propidium Iodide (PI) staining solution were sequentially introduced. Following an incubation duration of 15 min in the dark at room temperature, an additional 400 μL of 1 × Binding Buffer was added. Apoptosis rates (encompassing early Q4 and late Q2 apoptotic fractions) were determined via flow cytometry within 1 h.

### 2.12 Transmission electron microscopy

For ultrastructural identification of autophagic structures, transfected 12Z cells were cultured for 48 h, harvested via trypsinization, and immediately fixed with 2.5% glutaraldehyde (Solarbio, Beijing, China) at 4 °C overnight. The samples were then post-fixed in 1% osmium tetroxide, thoroughly rinsed, dehydrated through a graded series of ethanol solutions, and embedded in epoxy resin. Ultrathin sections (60–80 nm) were prepared using an ultramicrotome, followed by double electron staining with uranyl acetate and lead citrate. The characteristic autophagic vacuoles (autophagosomes and autolysosomes) were identified and imaged utilizing a transmission electron microscope.

### 2.13 Statistical analysis

All *in vitro* cell culture experiments were strictly performed in at least three independent biological replicates. For quantitative microplate-based assays, such as CCK-8 and RT-qPCR, appropriate technical replicates (e.g., triplicate wells) were also included within each biological run. Data were expressed as mean ± SD, and statistical significance was analyzed by Student’s t test (*p* < 0.05). Statistical analysis and graphics were performed using GraphPad Prism software (v9.0) and R software (v4.0.1).

## 3 Results

### 3.1 Screening and validation of the key gene (*CTSZ*) for endometriosis

**Figure 1:**
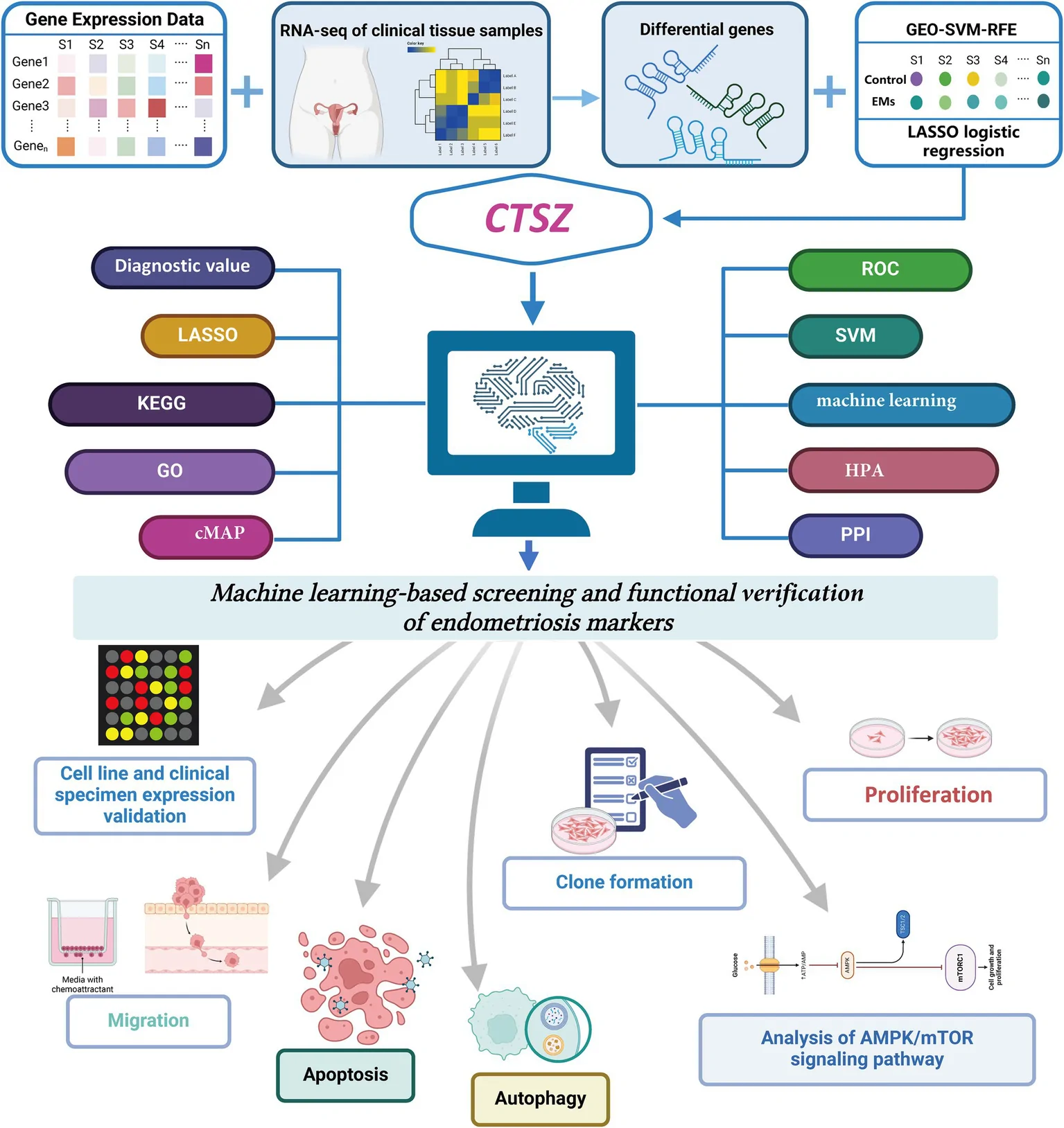
The main design and process of this study. The first step was the screening of the key pathogenic gene (CTSZ) of endometriosis and the verification of its expression level. The second step was the effect of knocking down CTSZ on the malignant biological function, apoptosis and autophagy of endometriosis cell lines. The third step was the verification and speculation of the potential regulatory pathways and small molecule therapeutic drugs of CTSZ.

**Figure 2:**
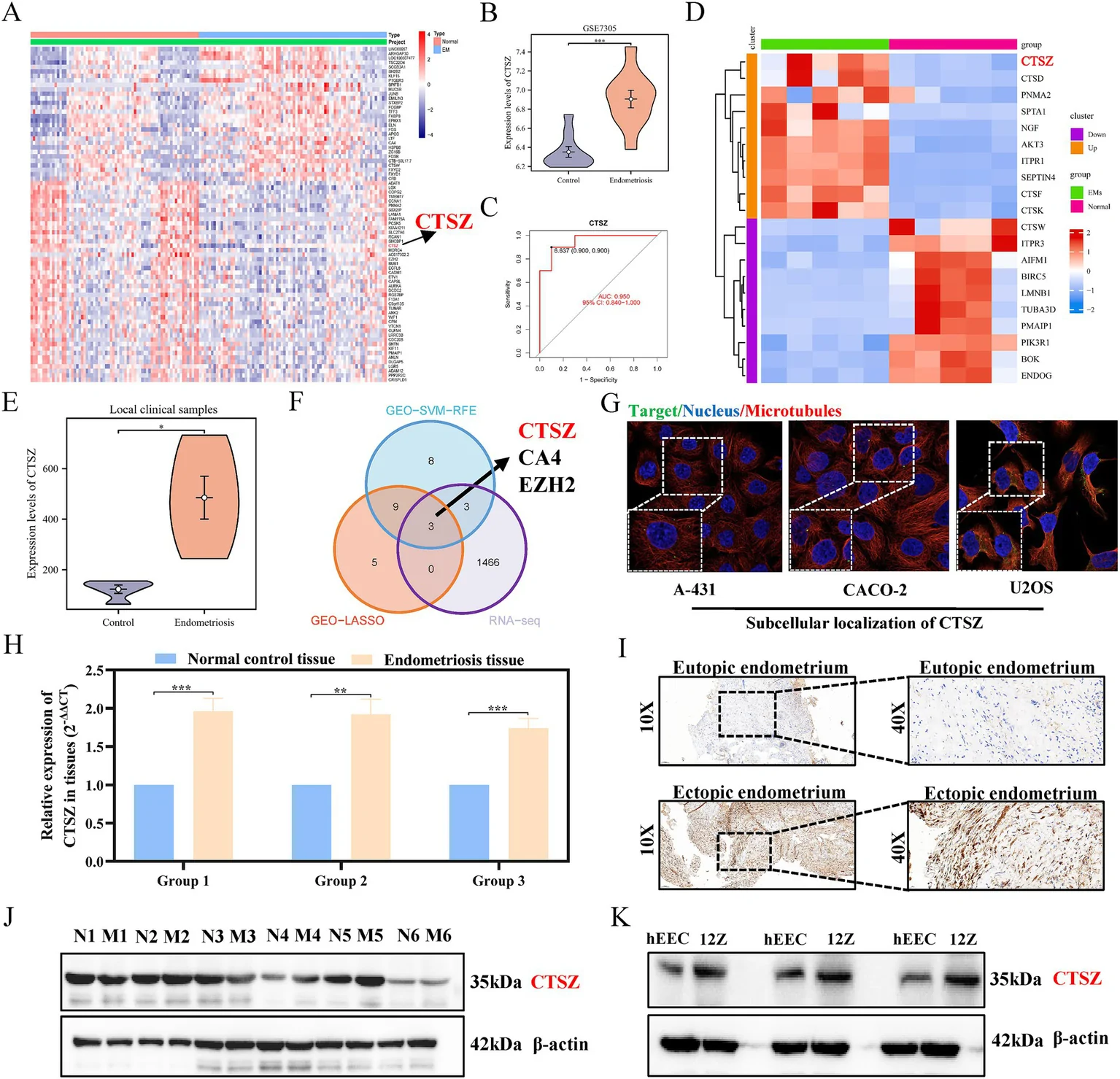
Screening and validation of the key pathogenic gene *CTSZ*. **(A)** Differential gene expression heatmap based on the GSE51981 dataset. **(B,C)** Expression level of *CTSZ* and diagnostic ROC curve based on the GSE7305 dataset. **(D,E)** Differential gene expression heatmap and relative expression level of *CTSZ* derived from RNA sequencing of local clinical tissue cohorts. **(F)** Identification of key pathogenic genes utilizing machine learning algorithms (LASSO and SVM-RFE). **(G)** Immunofluorescence subcellular localization of CTSZ obtained from the Human Protein Atlas (HPA). **(H)** Relative mRNA expression of *CTSZ* in normal control and endometriosis tissues determined by RT-qPCR. “Group 1, 2, and 3” denote three representative independent pairs of clinical tissue samples (Patient 1, 2, and 3). Error bars represent the standard deviation (SD) of three technical replicates per sample. **(I,J)** Protein expression levels and spatial localization of CTSZ in local clinical tissue samples evaluated via Western blotting and IHC. **(K)** Protein expression levels of CTSZ in cultured cell lines assessed via Western blotting and IHC.

The main experimental design and workflow of this study are illustrated in Figure 1. Initial exploration of the GSE51981 and GSE7305 datasets via gene expression heatmaps revealed distinct transcriptomic profiles in endometriosis (Figure 2A), with *CTSZ* being significantly overexpressed. Notably, ROC curve analysis demonstrated that *CTSZ* exhibited exceptional diagnostic value for the disease, achieving a remarkably high AUC of 0.950 (95% CI: 0.840–1.000) (Figures 2B,C). This upregulation was further corroborated by RNA sequencing of our local clinical cohort, which consistently identified *CTSZ* among the significantly upregulated differentially expressed genes (DEGs) in endometriosis tissues (Figures 2D,E). Subsequently, by integrating the public GEO datasets, local RNA sequencing data, and machine learning algorithms (LASSO and SVM-RFE), *CTSZ* was robustly screened out as a core pathogenic gene (Figure 2F). Furthermore, subcellular localization analysis via the Human Protein Atlas (HPA) supported the spatial distribution of CTSZ (Figure 2G).

To evaluate these bioinformatic findings experimentally, RT-qPCR was performed on local clinical samples, demonstrating the significant upregulation of *CTSZ* mRNA in endometriosis tissues (Figure 2H). Concurrently, elevated CTSZ protein expression in clinical tissues was evaluated through immunohistochemistry (IHC) and Western blotting (Figures 2I,J). Although individual clinical samples exhibited intrinsic physiological heterogeneity in baseline protein levels, comprehensive semi-quantitative analysis following normalization to *β*-actin revealed an overall upward trend of CTSZ protein expression in the majority of endometriosis tissues, which is highly consistent with the transcriptomic findings. *In vitro* assays further demonstrated the overexpression of CTSZ at the protein level across corresponding cultured cell lines (Figure 2K). Collectively, through the integration of transcriptomic sequencing and rigorous experimental validations, *CTSZ* was established as a key pathogenic gene in endometriosis, highlighting its potential as a diagnostic biomarker and therapeutic target.

### 3.2 Knockdown of *CTSZ* inhibited the malignant biological behaviors of endometriosis cell lines

Following the observation of *CTSZ* overexpression in endometriosis, we proceeded to knock down its expression to investigate its impact on the malignant biological behaviors of the endometriosis cell line (12Z). First, the knockdown efficiency of several siRNAs was evaluated via RT-qPCR and Western blotting. The si-429 sequence (hereafter referred to as si-*CTSZ*) demonstrated the most potent silencing efficacy (Supplementary Figure S1) and was consequently selected for subsequent functional assays.

**Figure 3:**
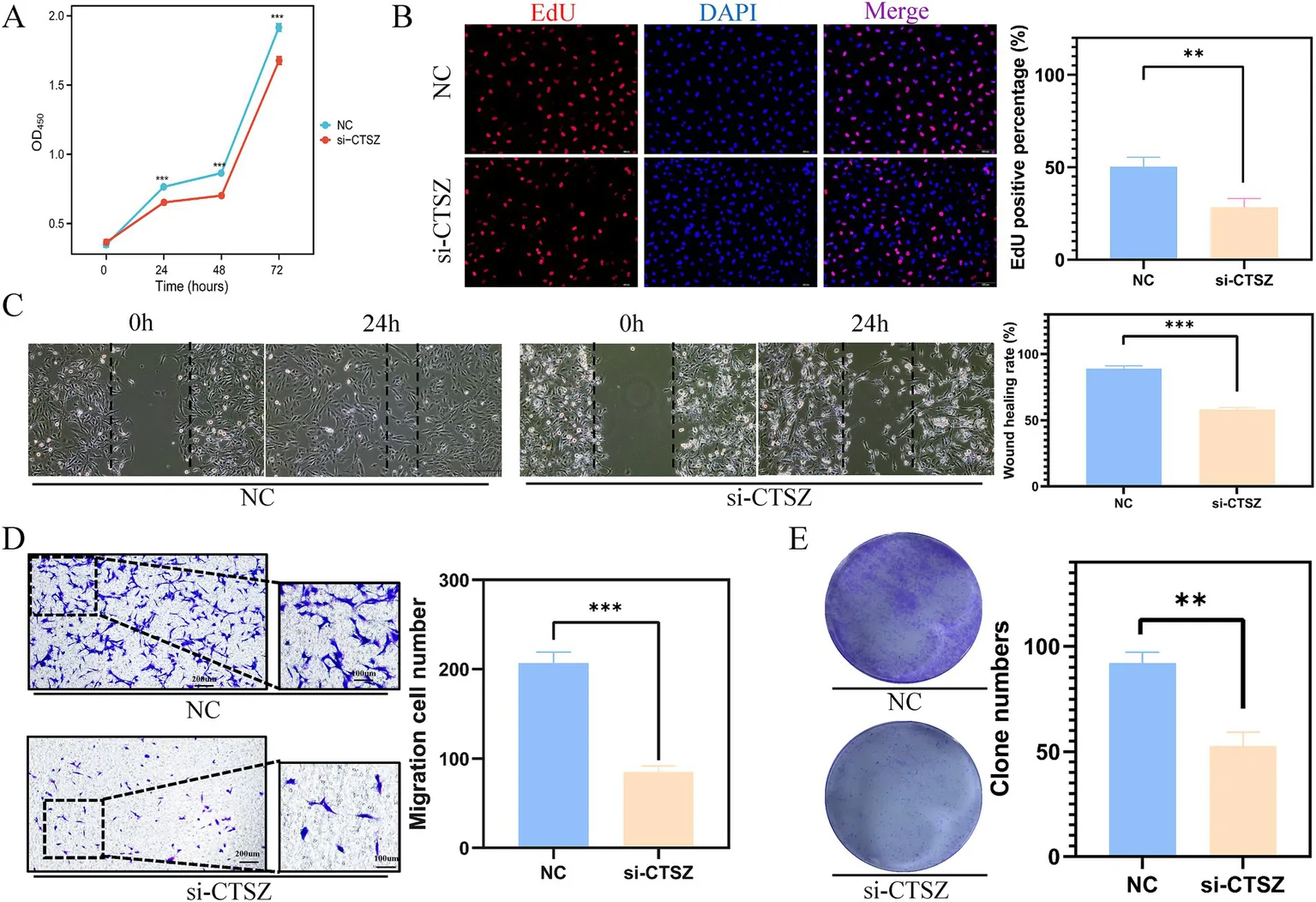
Effects of CTSZ knockdown on proliferation and migration. **(A)** Difference in absorbance (cell viability) between the CTSZ knockdown group (si-CTSZ) and the blank control group (NC), based on the CCK-8 assay. **(B)** Difference in the percentage of EdU-positive cells (cell proliferation) between the si-CTSZ group and the NC group, based on the EdU immunofluorescence assay. **(C)** Difference in the wound healing rate (cell migration) between the si-CTSZ group and the NC group, based on the wound healing assay. **(D)** Difference in the number of cells that migrated to the lower chamber of the Transwell (cell migration) between the si-CTSZ group and the NC group, based on the Transwell assay. **(E)** Difference in the number of clones formed (cell proliferation) between the si-CTSZ group and the NC group, based on the clonogenic assay.

Functional phenotypic analyses revealed that *CTSZ* depletion significantly impaired cell progression. The CCK-8 assay demonstrated a marked reduction in cell viability following *CTSZ* knockdown (Figure 3A). Concordantly, EdU immunofluorescence staining revealed a significant decrease in the percentage of EdU-positive cells in the si-*CTSZ* group, indicating an impaired proliferative capacity (Figure 3B). Furthermore, the wound healing assay showed a significantly delayed wound closure rate upon *CTSZ* silencing, suggesting an inhibition of horizontal migration (Figure 3C). This anti-migratory effect was further validated by the Transwell assay, wherein *CTSZ* depletion drastically reduced the number of cells invading the lower chamber (Figure 3D). Finally, the clonogenic assay indicated that *CTSZ* knockdown significantly impaired long-term colony-forming capabilities (Figure 3E). In summary, these *in vitro* experiments demonstrated that *CTSZ* silencing effectively suppresses the proliferation, migration, and colony formation of endometriosis cells.

### 3.3 Knockdown of CTSZ promoted apoptosis and autophagy in endometriosis cell lines

**Figure 4:**
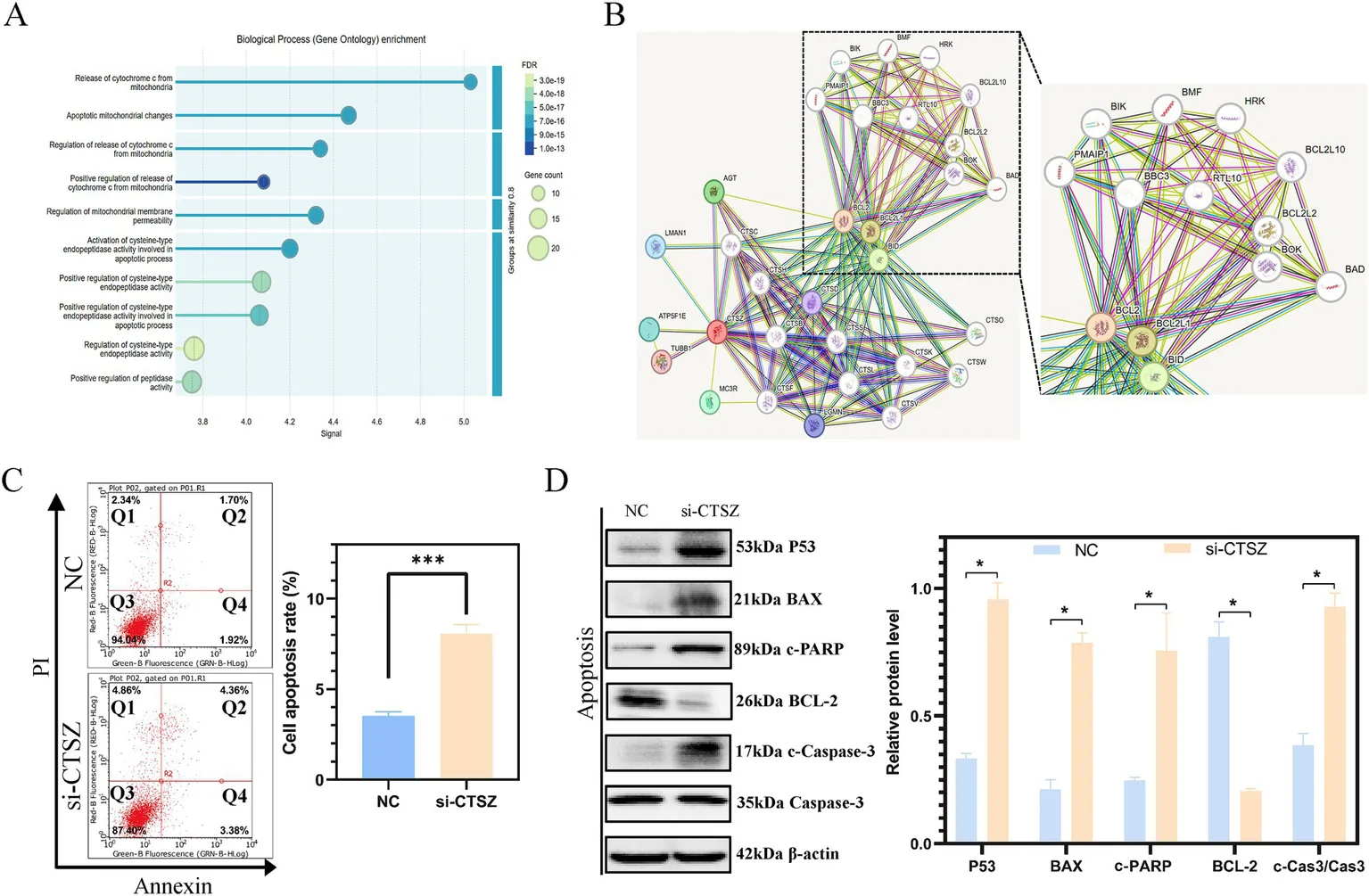
Effect of *CTSZ* knockdown on cell apoptosis. **(A)** Biological processes related to apoptosis enriched for *CTSZ*, analyzed using the public GEO dataset GSE51981 via GO analysis. **(B)** Potential protein–protein interaction (PPI) network of CTSZ associated with apoptosis, derived from the GSE51981 dataset. **(C)** Flow cytometry analysis of the apoptosis rate (Q2 + Q4: Late and early apoptotic cells) in the si-*CTSZ* and NC groups. **(D)** Protein expression levels of apoptosis-related markers (P53, BAX, PARP, BCL-2, Cleaved Caspase-3, and Caspase-3) in the si-*CTSZ* and NC groups evaluated via Western blotting.

After initially identifying the potential of CTSZ as a therapeutic target, considering the key regulatory ability of apoptosis and autophagy on endometriosis, we tried to explore the relationship between CTSZ and apoptosis and autophagy. Based on GO analysis, it was found that CTSZ may be enriched in apoptosis-related biological processes such as “Release of cytochrome c from mitochondria,” “Apoptotic mitochondrial changes,” “Regulation of release of cytochrome c from mitochondria,” “Regulation of mitochondrial membrane permeability” and “Activation of cysteine-type endopeptidase activity involved in apoptotic process” (Figure 4A). Based on PPI analysis, CTSZ may closely interact with apoptosis-related proteins such as BIK, BMF, HRK, PMAIP1, BBC3, BAD, BID, BOK, BCL2, BCL2L1, BCL2L2 and BCL2L10 (Figure 4B). Based on flow cytometry, it was observed that knockdown of CTSZ (si-CTSZ) could significantly increase the percentage of cells in late apoptotic state and early apoptotic state (Q2 + Q4), suggesting that it activated apoptosis in 12Z cell line (Figure 4C). Based on Western blotting, it was observed that knockdown of CTSZ (si-CTSZ) significantly upregulated the expression of apoptosis positive characteristic proteins (P53, BAX, PARP and c-Caspase3/Caspase3), and significantly downregulated the expression of apoptosis negative characteristic proteins (BCL-2), suggesting that it activated apoptosis in 12Z cell line (Figure 4D). Therefore, Figure 4 predicted that CTSZ is closely related to the biological process and proteins of apoptosis, and demonstrated that knocking down CTSZ could promote apoptosis in endometriosis cell lines.

**Figure 5:**
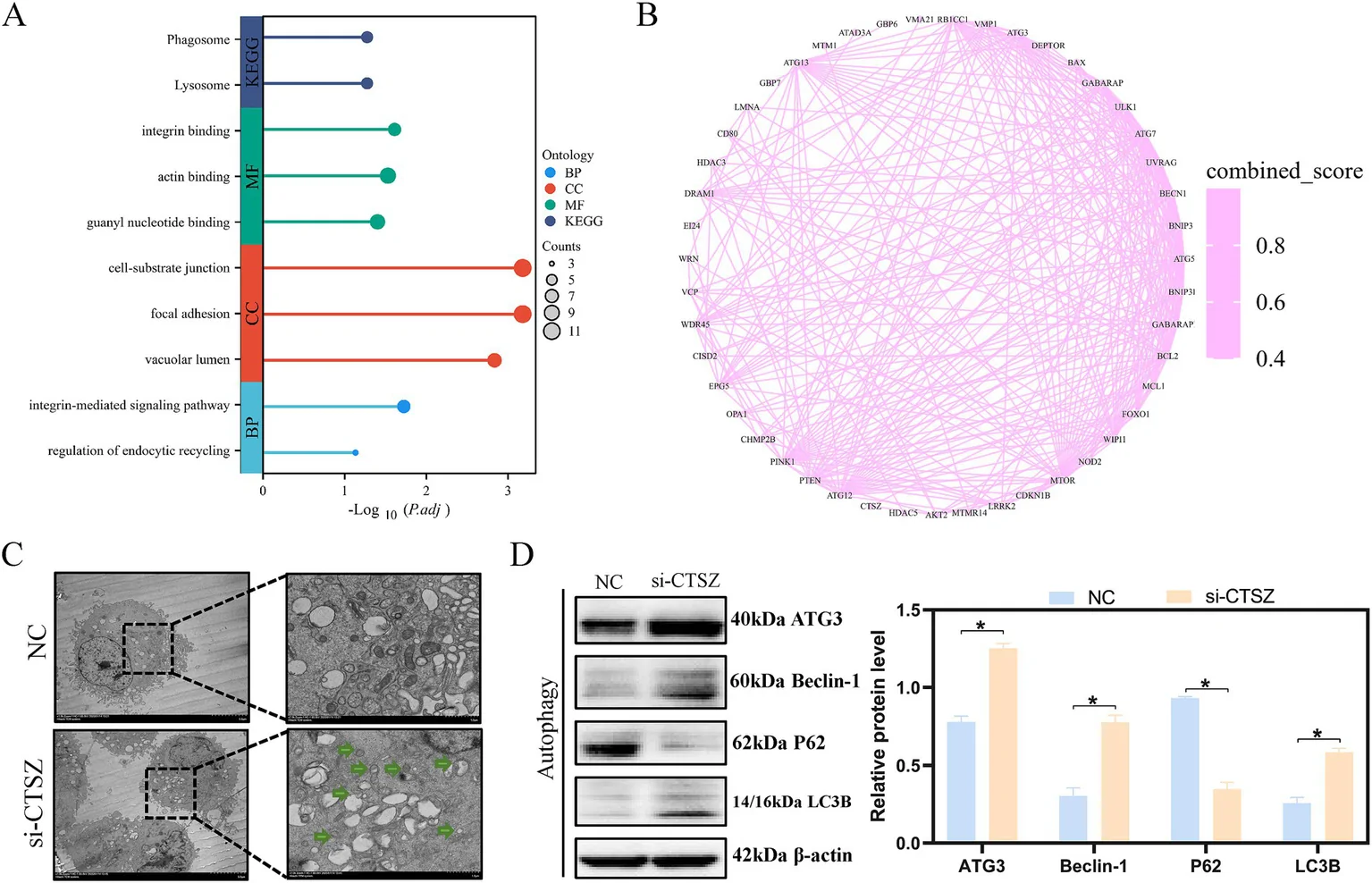
Effect of *CTSZ* knockdown on cell autophagy. **(A)** Biological processes related to autophagy enriched for *CTSZ*, analyzed using the GSE51981 dataset via GO analysis. **(B)** Potential protein–protein interaction (PPI) network of CTSZ associated with autophagy, derived from the GSE51981 dataset. **(C)** Representative transmission electron microscopy (TEM) images showing autophagy-related ultrastructures (e.g., autophagosomes) in the si-*CTSZ* and NC groups. **(D)** Protein expression levels of autophagy-related markers (ATG3, Beclin-1, P62, and LC3B) in the si-*CTSZ* and NC groups evaluated via Western blotting.

Next, the relationship between CTSZ and autophagy was explored. Based on GO analysis, it was found that CTSZ may be enriched in autophagy-related pathways such as “Phagosome,” “Lysosome,” “integrin binding,” “vacuolar lumen,” “and “regulation of endocytic recycling” (Figure 5A). Based on PPI analysis, CTSZ may closely interact with autophagy-related proteins such as ULK1, BECN1, ATG3, ATG5, ATG7, MTOR, PTEN, GABARAP, WIPI1, BNIP3 and PINK1 (Figure 5B). Based on transmission electron microscopy, it was found that knocking down CTSZ (si-CTSZ) could activate autophagy markers such as autophagosomes, suggesting that it activated autophagy in 12Z cell lines (Figure 5C). Based on Western blotting, it was demonstrated that knockdown of CTSZ (si-CTSZ) significantly upregulated the expression of autophagy positive characteristic proteins (ATG3, Beclin-1 and LC3B), and significantly downregulated the expression of autophagy negative characteristic protein (P62), suggesting that it activated autophagy in the 12Z cell line (Figure 4D). Therefore, Figure 5 predicted that CTSZ was closely related to the pathway and protein of autophagy, and supported that knockdown of CTSZ could promote autophagy in endometriosis cell lines.

### 3.4 Potential regulatory pathways and therapeutic drugs of CTSZ

**Figure 6:**
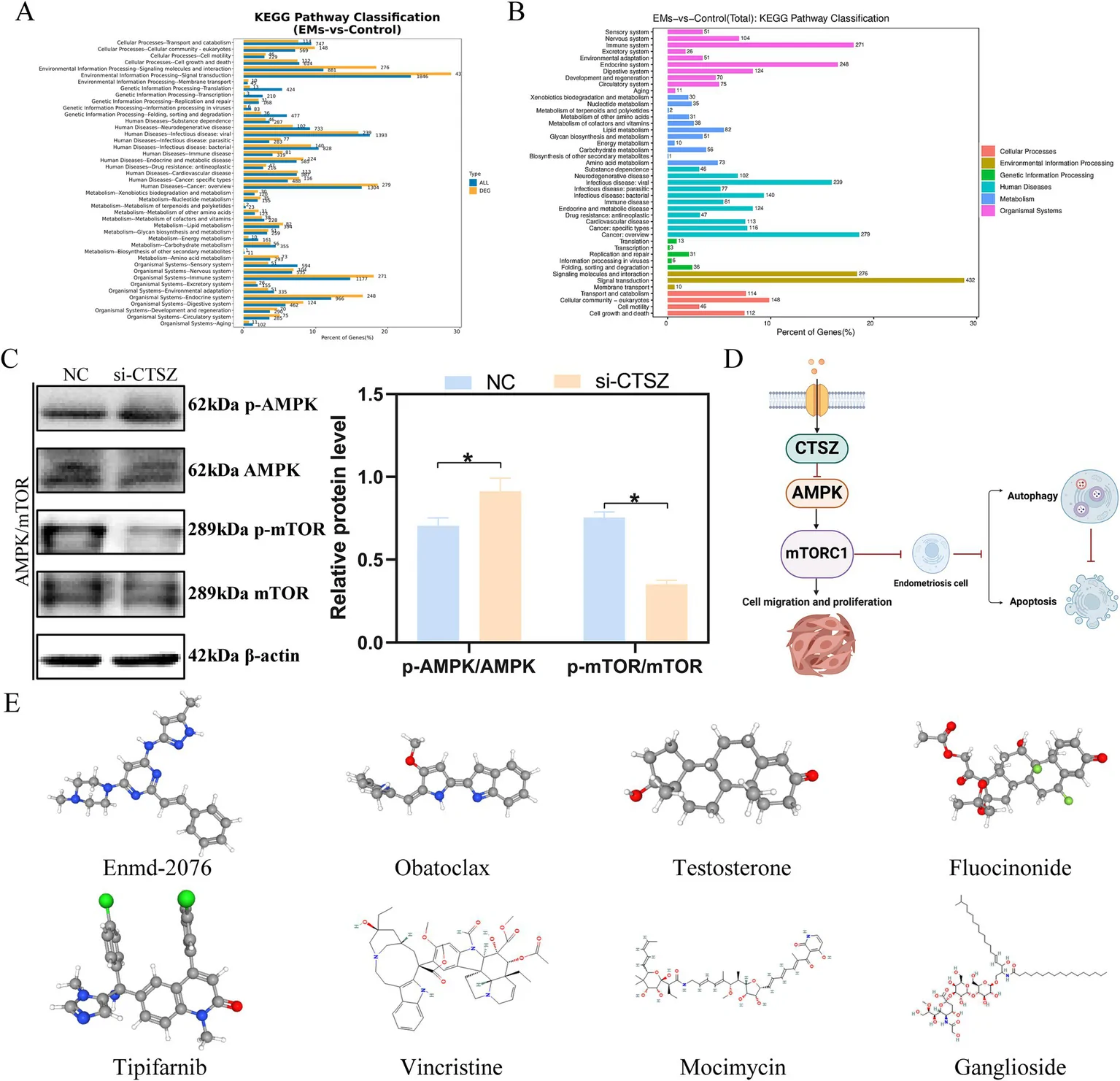
Potential regulatory pathways and therapeutic drugs targeting CTSZ. **(A,B)** Potential regulatory pathways enriched for *CTSZ*, analyzed using the GSE51981 dataset via KEGG analysis. **(C)** Protein expression levels of the AMPK/mTOR pathway markers (p-AMPK, AMPK, p-mTOR, and mTOR) in the si-*CTSZ* and NC groups evaluated via Western blotting. **(D)** Schematic diagram speculating the potential regulatory mechanisms of *CTSZ* in endometriosis. **(E)** The 3D chemical structures and scientific names of the eight candidate therapeutic drugs targeting the CTSZ network. Detailed specifications for these compounds are provided in Table 1.

After preliminarily evaluating the regulatory effects of CTSZ on apoptosis and autophagy, this study attempted to explore deeper potential regulatory pathways. Based on KEGG analysis, it was found that CTSZ may be enriched in regulatory pathways such as “Signal transduction,” “Transport and catabolism,” “Energy metabolism” and “Carbohydrate metabolism” (Figures 6A,B). After comprehensively considering the relationship between these pathways, endometriosis, apoptosis and autophagy, this study speculated that CTSZ may be related to the AMPK/mTOR pathway, and then conducted relevant verification. Based on Western blotting, it was demonstrated that knockdown of CTSZ (si-CTSZ) significantly upregulated the ratio of p-AMPK/AMPK and significantly downregulated the ratio of p-mTOR and mTOR, suggesting that it activated AMPK and inhibited mTOR, that is, knockdown of CTSZ could regulate the AMPK/mTOR pathway (Figure 6C). Therefore, considering the relationship between CTSZ, autophagy, apoptosis, AMPK/mTOR pathway and endometriosis, combined with the experimental results of this study, this study could make a reasonable speculation that CTSZ may be able to inhibit AMPK, activate mTOR, and then inhibit autophagy and synergistically inhibit apoptosis, and finally promote the proliferation and migration of endometriosis cell lines and other malignant biological behaviors (Figure 6D).

The above findings suggested that CTSZ could be used as an important potential therapeutic target for endometriosis. So, how to use this target as an entry point for treatment? Based on cMAP analysis, eight candidate small-molecule therapeutic drugs that may target the CTSZ network were identified. The 3D chemical conformations of these candidates are visualized in Figure 6E, while their detailed specifications—including PubChem CIDs, molecular formulas, and corresponding pharmacological mechanisms—are comprehensively summarized in Table 1. In summary, the key gene CTSZ could not only serve as a potential biomarker, but also promote malignant progression through key pathways such as AMPK/mTOR/autophagy/apoptosis. It is also likely to serve as a therapeutic target for the above-mentioned small molecule drugs and play a potential therapeutic role in endometriosis.

**Table 1:** Candidate therapeutic drugs targeting the CTSZ network for endometriosis.

Candidate drug	PubChem CID	Molecular formula	Pharmacological mechanism and potential role in endometriosis
Enmd-2076	16,041,424	C_21_H_25_N_7_	Multi-targeted kinase inhibitor (Exhibits potent anti-angiogenic and anti-proliferative activities to potentially restrict endometriotic lesion growth and neovascularization).
Obatoclax	45,356,963	C_20_H_19_N_3_O	Apoptosis inducer/BH3 mimetic (Overcomes apoptosis resistance in ectopic lesions by targeting the BCL-2 protein family).
Testosterone	6,013	C_19_H_28_O_2_	Androgen receptor agonist (Modulates hormone receptor expression; potentially intervenes in progesterone resistance to restrict endometrial proliferation).
Fluocinonide	9,642	C_26_H_32_F_2_O_7_	Glucocorticoid receptor agonist (Potent anti-inflammatory agent; targets the chronic inflammatory microenvironment typical of endometriosis).
Tipifarnib	159,324	C_27_H_22_Cl_2_N_4_O	Farnesyltransferase inhibitor (Modulates key intracellular signaling pathways, such as Ras, potentially limiting abnormal cell proliferation and survival).
Vincristine	5,978	C_46_H_56_N_4_O_10_	Microtubule destabilizer/Mitotic inhibitor (Suppresses the hyperproliferative capacity of endometriotic epithelial cells).
Mocimycin	135,484,176	C_43_H_60_N_2_O_12_	Antibacterial/Elongation factor Tu inhibitor (Potential immunomodulator; may alter the pelvic microbiome and inflammatory milieu).
Ganglioside	163,110,884	C_59_H_108_N_2_O_22_	Neuromodulator/Sphingolipid (Involved in lipid metabolism and neural signaling; potentially targets neuroinflammation and endometriosis-associated pelvic pain).

## 4 Discussion

The key biomarkers, therapeutic targets and pathological mechanisms of endometriosis need to be elucidated (1, 4). Cathepsins, especially CTSZ, are closely related to key features of EM, such as inflammation and angiogenesis (11, 17). Autophagy and apoptosis may promote the occurrence and development of EM through lysosomes and may be regulated by CTSZ (22, 24). Therefore, this study attempts to clarify the role of CTSZ in EM, explore the potential regulatory mechanism of CTSZ and corresponding therapeutic drugs, and demonstrate the feasibility of CTSZ as a biomarker and therapeutic target, ultimately improving the diagnosis and treatment dilemma of EM.

Screening and validating the key genes of EM was the first step of this study. Figure 2 first screened out the key pathogenic gene (*CTSZ*) of EM based on public data, local clinical sample sequencing and basic experiments, and solidly demonstrated the abnormally high expression of *CTSZ*, an important member of the cathepsin family. The dysregulation of cathepsins is inseparable from the occurrence and development of EM (13–15). In addition, some studies have used cathepsins as therapeutic targets to delay the disease progression of EM by inhibiting them (16). While previous studies have primarily linked other family members (such as CTSB and CTSG) to basic extracellular matrix degradation, our findings highlight a distinct and more complex intracellular signaling role for our protagonist, CTSZ. It has also been found to be closely involved in malignant proliferation, inflammation, angiogenesis and other processes, which are also characteristic processes of EM (17, 18, 20). Afterwards, Figure 3 indicated that knocking down CTSZ could inhibit the proliferation and migration of EM and other malignant behaviors based on a series of solid cytological experiments. Some studies have also suggested that CTSZ not only promotes the migration and invasion of T lymphocytes (26), but also promotes the malignant proliferation and migration of colorectal cancer (27). Therefore, combined with the findings in Figures 2,3 of this study and related literature, it is reasonable to speculate that as a pathogenic gene, abnormally high expression of CTSZ could promote malignant proliferation and migration, thereby promoting the disease progression of EM. However, the specific regulatory mechanism is still unclear.

The pathology of EM is extremely complex, but the dysregulation of apoptosis and autophagy must be an important part of it (22, 23, 28). First, apoptosis was used as the starting point for exploration. Figure 4A found that CTSZ may be enriched in biological processes such as apoptosis, changes in mitochondrial membrane permeability, and cytochrome C release. As we all know, changes in mitochondrial membrane permeability and cytochrome C release are closely related to apoptosis (29, 30). Figure 4B found that CTSZ may interact with proteins such as BCL2, BCL2L1, BMF, BIK, BID, and BOK. These BCL2 family proteins and related proteins profoundly regulate apoptosis, the important biological process (31, 32). And BMF protein may also play a key role in EM-related pain perception and maintenance (33). Figures 4C,D strongly supported that knocking down CTSZ could promote apoptosis. Studies have shown that CTSZ, a key therapeutic target, could be inhibited by PSCD4, thereby promoting apoptosis in multiple myeloma and playing a potential therapeutic role (34). Therefore, combined with the findings of Figure 4 of this study and related literature, it could be reasonably speculated that CTSZ, a key pathogenic gene, may worsen EM by inhibiting apoptosis.

Next, we will continue to explore autophagy (23). Figure 5A showed that CTSZ may be enriched in autophagy-related biological processes such as lysosomes, phagosomes, and endocytosis (35). Figure 5B showed that CTSZ may interact with autophagy-related proteins such as ULK1, BECN1, ATG3, mTOR, and PINK1 (36). Studies have shown that inhibition of mTOR could induce autophagy and promote cell apoptosis, thereby treating EM (37). Figures 5C,D strongly supported that knocking down CTSZ could induce autophagy. Interestingly, studies have shown that the stability of lysosomes could be destroyed by a specific drug B10, which in turn regulates CTSZ and induces autophagy and apoptosis (24). Importantly, the inhibition of autophagy could regulate sex hormone synthesis and inhibit apoptosis, ultimately exacerbating the progression of EM (22, 23). Therefore, combined with the findings of Figure 5 of this study and related literature, it is reasonable to speculate that CTSZ, a key malignant gene, may inhibit autophagy and thus synergistically inhibit apoptosis, ultimately worsening EM.

After exploring apoptosis and autophagy, this study attempted to explore deeper potential mechanisms. Figures 6A,B found that CTSZ may be related to signal transduction, energy metabolism, carbohydrate metabolism, transport and catabolism. It is well known that the AMPK/mTOR signaling pathway, as a cell metabolism and signal integration center, is closely related to the above pathways (38, 39). Moreover, Figure 5B of this study also found that CTSZ may interact closely with mTOR. Therefore, this study decided to continue to explore the further in-depth regulatory mechanism of CTSZ with the AMPK/mTOR signaling pathway as the entry point. Figure 6C demonstrated that knocking down *CTSZ* could activate AMPK and inhibit mTOR, thereby modulating the AMPK/mTOR signaling axis. The dysregulation of the AMPK/mTOR signaling pathway is crucial in the occurrence, development, and metastasis of EM (40, 41). Therefore, combined with the findings of this study and related literature, it could be reasonably speculated that CTSZ may exert its pathogenic role by suppressing AMPK and activating mTOR, thereby inhibiting autophagy and synergistically inhibiting apoptosis, and ultimately promoting the proliferation and migration of endometriosis cell lines and other malignant biological behaviors (Figure 6D). In addition, in order to achieve the goal of diagnosis and treatment synergy in this study, potential therapeutic drugs were also explored. As visualized in Figure 6E and mechanistically detailed in Table 1, we identified specific small-molecule therapeutic drugs that may target CTSZ, including Enmd-2076, Obatoclax, Testosterone, and Fluocinonide. Enmd-2076 was found to be related to key features of endometriosis such as angiogenesis and tumor growth (42, 43). Obatoclax, as an apoptosis inducer, could activate apoptosis and thus treat endometriosis, which is consistent with this study in some aspects (44, 45). Testosterone may intervene in progesterone resistance by upregulating the expression of progesterone receptors, thereby regulating the growth of the endometrium (46, 47). Endometriosis is a typical chronic inflammatory disease, and Fluocinonide, a glucocorticoid, could play an indispensable therapeutic role in inflammatory diseases (48). Therefore, combined with the findings of Figure 6 of this study and related literature, the logical cascade of disease suppression can be systematically connected: Abnormally high *CTSZ* suppresses AMPK and activates mTOR signaling, thereby blunting protective autophagy and apoptosis to fuel disease progression. Conversely, targeting and knocking down *CTSZ* restores AMPK activation and subsequently inhibits mTOR. This pivotal signaling shift triggers a robust induction of autophagy, which, synergistically with apoptosis, dismantles the proliferative and migratory capacities of endometriotic cells, ultimately resulting in profound disease suppression. Furthermore, *CTSZ* may serve as a promising therapeutic target for the aforementioned small molecule drugs to improve the current clinical management of endometriosis. Collectively, these mechanistic insights add crucial new knowledge to the existing literature. They emphasize the unique biological role of CTSZ not merely as a conventional downstream protease, but as a critical upstream metabolic modulator. By uncovering the previously unrecognized CTSZ-AMPK-mTOR-autophagy/apoptosis axis, our study provides a novel and comprehensive pathophysiological network for EM.

Despite the promising translational potential of CTSZ identified in this study, several limitations should be explicitly acknowledged. First, the *in vitro* functional mechanisms of *CTSZ* were primarily explored through a loss-of-function strategy in a single representative endometriotic epithelial cell line (12Z). This decision was driven by the primary focus of our assays on cellular migration and proliferation—phenotypes critically mediated by the endometriotic epithelium during initial lesion adhesion and invasion. Furthermore, considering its function as a cysteine proteinase, CTSZ is closely linked to extracellular matrix degradation, making the highly active epithelial model biologically appropriate for observing its migratory effects. While our clinical cohort validations align with these *in vitro* observations, future studies should incorporate additional endometriotic cell lines and normal endometrial epithelial models with bi-directional modulations (e.g., overexpression) to further solidify its multi-dimensional roles. Second, as endometriosis involves a highly complex microenvironment, utilizing only epithelial cells precludes the evaluation of critical cross-talks with endometrial stromal cells (EESCs) and immune infiltrates. Future investigations utilizing sophisticated 3D co-culture systems or patient-derived organoids are warranted to comprehensively map the pathophysiological network of CTSZ. Third, owing to the relatively limited sample size of our local clinical validation cohort, we were unable to perform a robust statistical correlation analysis linking CTSZ expression levels to detailed clinical parameters, such as ASRM staging, the severity of dysmenorrhea (pelvic pain), and long-term post-operative recurrence rates. Future multi-center studies with expanded, well-annotated clinical cohorts are essential to firmly establish the clinical and prognostic utility of CTSZ. Fourth, our current data linking CTSZ to the AMPK/mTOR signaling is primarily based on associative protein expression profiling. To establish definitive mechanistic causality, subsequent studies utilizing specific pathway modulators (e.g., Compound C, AICAR, or Rapamycin) are imperative. Finally, although we speculated on potential therapeutic drugs targeting the CTSZ protein via bioinformatics, these candidates lack empirical validation. In the future, utilizing specific CTSZ inhibitors and the aforementioned therapeutic drugs as entry points in *in vivo* models will be the primary focus of our ongoing research, aiming to further expand the translational progress in endometriosis treatment.

## 5 Conclusion

In conclusion, this study contributes novel insights to the existing literature by uncovering the unique biological role of CTSZ as a critical upstream metabolic modulator in endometriosis, distinguishing it from traditional extracellular proteases. *CTSZ* acts as a key pathogenic gene, and its knockdown could activate AMPK and subsequently inhibit mTOR signaling, thereby inducing autophagy and synergistically promoting apoptosis to dismantle the proliferative and migratory capacities of endometriotic cells. By mapping this previously unrecognized CTSZ-AMPK-mTOR pathophysiological network, our findings establish CTSZ as a promising diagnostic biomarker and a viable target for novel therapeutic interventions.

## Data Availability

The datasets presented in this study can be found in online repositories. The names of the repository/repositories and accession number(s) can be found in the article/Supplementary material.

## Glossary

EM: Endometriosis
CTSZ: Cathepsin Z
CTSB: Cathepsin B
CTSG: Cathepsin G
GEO: Gene Expression Omnibus
RNA-seq: RNA sequencing
LASSO: Least Absolute Shrinkage and Selection Operator
SVM-RFE: Support Vector Machine-Recursive Feature Elimination
PPI: Protein–Protein Interaction networks
HPA: Human Protein Atlas
IHC: Immunohistochemistry
Go: Gene Ontology
KEGG: Kyoto Encyclopedia of Genes and Genomes
cMAP: Connectivity Map
hEEC: human immortalized endometrial cells
12Z: Human immortalized endometriosis cells
P53: Tumor protein 53
BAX: BCL2-associated X protein
PARP: Poly(ADP-ribose) polymerase
BCL-2: B-cell lymphoma 2
Caspase-3: Cysteine-aspartic protease 3
c-Caspase-3: Cleaved Caspase-3)
ATG3: Autophagy Related Gene 3
Beclin-1: BECN1
P62: Sequestosome 1
LC3B: Microtubule Associated Protein 1 Light Chain 3 Beta
p-AMPK: Phosphorylated AMP-activated Protein Kinase
AMPK: AMP-activated Protein Kinase
p-mTOR: Phosphorylated Mechanistic Target of Rapamycin
mTOR: Mechanistic Target of Rapamycin.
