# Supplementary material for: Targeting Cathepsin Z suppresses endometriosis via the AMPK/mTOR-mediated autophagy and apoptosis axis
Source: Front Med (Lausanne). 2026 Sep 1;13:1681467. doi: 10.3389/fmed.2026.1681467 (PMC13574666; doi:10.3389/fmed.2026.1681467)
Supplement: Supplementary file 1 [file Supplementary_file_1.docx]

**Supplementary Material**

**Title: Targeting Cathepsin Z Suppresses Endometriosis via the AMPK/mTOR-Mediated Autophagy and Apoptosis Axis**

**Running Title:** **CTSZ: Biomarker and Therapeutic Target in Endometriosis**

Mengjun Zhang ^1&^, Jialin Wang ^4&^, Haodi Yue ^3&^, Zidi Zhang ^1^, Lindong Zhang ^1@^ and Xin Zhao ^2,5@^

1 Department of Gynecology, The Third Affiliated Hospital of Zhengzhou University, 7 Rehabilitation Front Street, Zhengzhou 450052, China.

2 Department of Radiology, The Third Affiliated Hospital of Zhengzhou University, 7 Rehabilitation Front Street, Zhengzhou 450052, China.

3 Department of Center for Clinical Single Cell Biomedicine, Henan Provincial People's Hospital, People's Hospital of Zhengzhou University, No. 7 Weiwu Street, Zhengzhou 450003, China.

4 Department of Orthopedics, The First Affiliated Hospital of Zhengzhou University, No.1, East Jianshe Road, 450052, Zhengzhou, China.

5 Tianjian Laboratory of Advanced Biomedical Sciences, Institute of Advanced Biomedical Sciences, Zhengzhou University, Zhengzhou 450052, China.

Mengjun Zhang ^1&^, Jialin Wang ^4&^ and Haodi Yue ^3&^ were the co-first authors.

Lindong Zhang ^1@^ and Xin Zhao ^2,5@^ were the co-corresponding authors.

**Corresponding Author:**

Xin Zhao ^2,5@^, Ph.D.,

Department of Radiology, The Third Affiliated Hospital of Zhengzhou University, 7 Rehabilitation Front Street, Zhengzhou 450052, China.

Tianjian Laboratory of Advanced Biomedical Sciences, Institute of Advanced Biomedical Sciences, Zhengzhou University, Zhengzhou 450052, China.

E-mail: zdsfyzx@zzu.edu.cn

Lindong Zhang ^1@^, Ph.D.,

Department of Gynecology, The Third Affiliated Hospital of Zhengzhou University, 7 Rehabilitation Front Street, Zhengzhou 450052, China.

E-mail: zld8399@163.com

**Contents:**

**Table S1. Detailed specifications of primary and secondary antibodies and reagents utilized in this study.**

**Figure S1. Evaluation of CTSZ knockdown efficiency by different siRNAs.**

**Table S1. Detailed specifications of primary and secondary antibodies and reagents utilized in this study.**

| No. | Antibody /  Reagent | Host Species | Application | Dilution | Manufacturer | Catalogue No. |
| --- | --- | --- | --- | --- | --- | --- |
| 1 | CTSZ | Rabbit | IHC, WB | 1:1000 | Affinity Biosciences, China | DF14386 |
| 2 | P53 | Mouse | WB | 1:1000 | Cell Signaling Technology, USA | #2524 |
| 3 | BAX | Rabbit | WB | 1:1000 | Cell Signaling Technology, USA | #2772 |
| 4 | BCL-2 | Rabbit | WB | 1:1000 | Affinity Biosciences, China | AF6139 |
| 5 | Cleaved-PARP | Rabbit | WB | 1:1000 | Affinity Biosciences, China | AF7023 |
| 6 | Caspase-3 | Rabbit | WB | 1:1000 | Cell Signaling Technology, USA | #9662 |
| 7 | Cleaved Caspase-3 | Rabbit | WB | 1:1000 | Cell Signaling Technology, USA | #9661 |
| 8 | ATG3 | Rabbit | WB | 1:1000 | Affinity Biosciences, China | DF8216 |
| 9 | Beclin-1 | Rabbit | WB | 1:1000 | Cell Signaling Technology, USA | #3738 |
| 10 | P62 | Mouse | WB | 1:1000 | Cell Signaling Technology, USA | #88588 |
| 11 | LC3B | Rabbit | WB | 1:1000 | Cell Signaling Technology, USA | #2775 |
| 12 | p-AMPK | Rabbit | WB | 1:1000 | Cell Signaling Technology, USA | #2537 |
| 13 | AMPK | Rabbit | WB | 1:1000 | Cell Signaling Technology, USA | #2795 |
| 14 | p-mTOR | Rabbit | WB | 1:1000 | Cell Signaling Technology, USA | #5536 |
| 15 | mTOR | Rabbit | WB | 1:1000 | Cell Signaling Technology, USA | #2972 |
| 16 | β-actin | Mouse | WB | 1:10000 | Affinity Biosciences, China | T0022 |
| 17 | HRP -linked Anti-Rabbit IgG | Goat | WB, IHC | 1:3000 | Cell Signaling Technology, USA | #7074 |
| 18 | HRP -linked Anti-Mouse IgG | Horse | WB | 1:3000 | Cell Signaling Technology, USA | #7076 |
| 19 | SuperPico ECL Chemiluminescence Kit | N/A | WB | Ready-to-use | Vazyme, China | E422-01 |

**Figure S1. Evaluation of CTSZ knockdown efficiency by different siRNAs.**

(A) Western blot analysis of CTSZ protein levels in 12Z cells following transfection with blank control, negative control (NC), and three distinct siRNAs (si-635, si-429, and si-727). β-actin was utilized as the endogenous loading control. (B) Quantitative densitometry analysis of relative CTSZ protein expression levels normalized to β-actin. (C) Relative CTSZ mRNA expression levels evaluated by RT-qPCR in different transfection groups. Data are presented as mean ± SD from three independent experiments (***P < 0.001, **P < 0.01). The si-727 construct exhibited the highest silencing efficacy and was thus selected for all subsequent functional downstream assays.
